# Population Pharmacokinetic Analysis of Amikacin for Optimal Pharmacotherapy in Korean Patients with Nontuberculous Mycobacterial Pulmonary Disease

**DOI:** 10.3390/antibiotics9110784

**Published:** 2020-11-06

**Authors:** Xuanyou Jin, Jaeseong Oh, Joo-Youn Cho, SeungHwan Lee, Su-jin Rhee

**Affiliations:** Department of Clinical Pharmacology and Therapeutics, Seoul National University College of Medicine and Hospital, Seoul 03080, Korea; jsenu@snu.ac.kr (X.J.); johan25@snu.ac.kr (J.O.); joocho@snu.ac.kr (J.-Y.C.); leejh413@snu.ac.kr (S.L.)

**Keywords:** nontuberculous mycobacterial pulmonary disease, amikacin, population pharmacokinetics

## Abstract

Amikacin is used as a therapy for patients with nontuberculous mycobacterial pulmonary disease (NTM-PD) who are resistant to macrolide antibiotics or have severe symptoms. This study aimed to characterize the pharmacokinetic properties of amikacin in patients with NTM-PD by developing a population pharmacokinetic model and to explore the optimal pharmacotherapy in patients with NTM-PD. For this study, all data were retrospectively collected. The amikacin pharmacokinetic properties were best described by a two-compartment model with first-order elimination. The estimated glomerular filtration rate and body weight were identified as significant covariates for clearance and the volume of distribution, respectively. A model-based simulation was conducted to explore the probability of reaching the target therapeutic range when various dose regimens were administered according to the body weight and renal function. The simulation results indicated that the amikacin dosage should be determined based on the body weight, and for patients who weigh over 70 kg, it is necessary to adjust the dose according to renal function. In conclusion, the optimal pharmacotherapy of amikacin for patients with NTM-PD was recommended based on the population pharmacokinetic model, which is expected to enable the personalization of drug therapy and improve the clinical outcomes of amikacin therapy.

## 1. Introduction

Nontuberculous mycobacterial pulmonary disease (NTM-PD) is a chronic pulmonary disease caused by infection with nontuberculous mycobacteria (NTM) [1]. NTM are also known as atypical mycobacteria, which are mycobacteria other than those that cause tuberculosis, and are normally present in the environment [2]. NTM can produce clinical diseases in any body tissue, such as pulmonary and lymphatic and skin/soft tissue, and the most common manifestation of NTM disease is pulmonary disease [3]. Macrolide antibiotics form the basis of therapy for NTM-PD, and patients with macrolide resistance or severe NTM-PD are treated with amikacin [4].

Amikacin is an aminoglycoside antibiotic that is used for the treatment of severe infections caused by multidrug-resistant, aerobic Gram-negative bacteria that bind to bacterial 30S ribosomal subunits and leads to the disruption of normal protein synthesis and the production of nonfunctional or toxic peptides [5]. According to the mechanism of action, amikacin is used for the treatment of NTM-PD, and the recommended dose is 10–15 mg/kg once daily for patients with NTM infection [4]. Meanwhile, aminoglycosides including amikacin are known to have low penetration into lung tissue, so a higher therapeutic concentration may be required for the treatment of pulmonary disease than other indications.

Amikacin exhibits linear pharmacokinetic (PK) properties in the range of 1.5–20 mg/kg doses, and it has been reported that the volume of distribution is 0.21 L/kg, the clearance is approximately 94 mL/min with normal renal function, and the half-life is approximately 2 h. The vast majority of amikacin is secreted unchanged via glomerular filtration, and less than 11% of the administered dose of amikacin actually binds to plasma proteins [6,7]. Amikacin showed concentration-dependent killing of bacteria, and the therapeutic target ranged from 8 to 10 as a ratio of the peak concentration (C_peak_) to the minimal inhibitory concentration (MIC) of the bacteria [8,9]. The utility of amikacin is limited owing to its well-known adverse effects, such as nephrotoxicity and ototoxicity. The occurrence of amikacin adverse events is associated with a variety of factors, including the C_peak_, trough serum concentration (C_trough_), treatment duration, and cumulative dose [5]. Therefore, in general, to achieve sufficient effects while reducing toxicity, the guidelines recommend that the C_peak_ should be adjusted to less than 35 µg/mL and that the C_trough_ should be less than 10 µg/mL [10]. However, for the treatment of NTM-PD, the target concentration is higher than that for other indications; the guidelines recommend that the C_peak_ should be adjusted to 35–45 µg/mL with once-daily administration and that the C_trough_ should be maintained at less than 4 µg/mL [10,11]. Despite these guidelines, several studies indicated that amikacin toxicity was observed in 3.3–40.2% of patients with NTM-PD [12,13,14].

The prevalence and incidence of NTM-PD have increased globally, including in Korea [15,16,17]. Due to the toxicity and narrow therapeutic range of amikacin, more specific and accurate dosage recommendations are needed. The PK profile of amikacin has not been evaluated in patients with NTM-PD. Therefore, the aims of this study were to evaluate the PK properties of amikacin in patients with NTM-PD by using population PK analysis and to explore the regimen necessary for target attainment through a model-based simulation.

## 2. Results

### 2.1. Demographic Characteristics

In total, 848 serum amikacin concentration-time data points were collected from 70 adult patients who received amikacin for the treatment of NTM-PD. Of the analyzed patients, 51 (73%) were females, and 19 (27%) were males. The patients were between 25 and 85 years of age, and the body weight ranged from 29.9 to 79.8 kg (Table 1).

### 2.2. Final Population Pharmacokinetic Model

**Figure 1:**
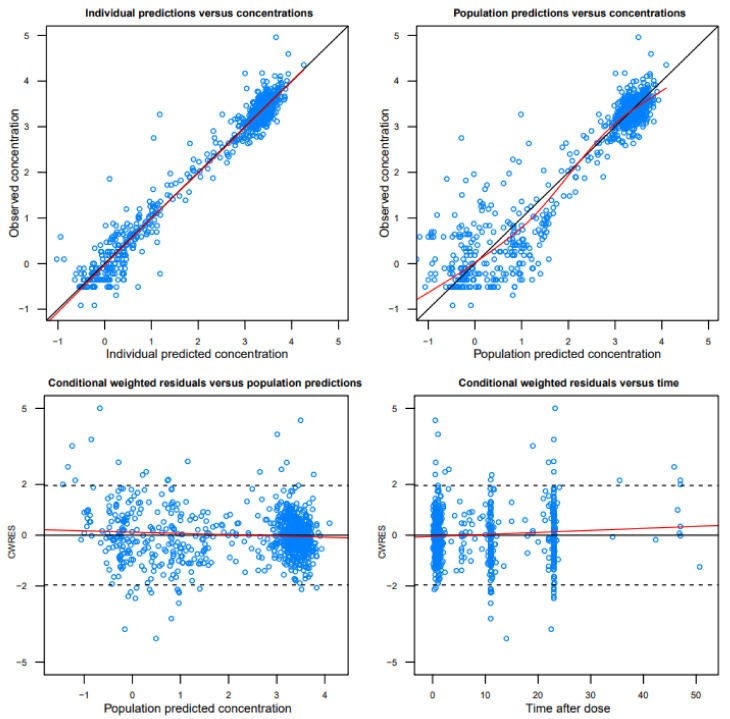
Goodness-of-fit plots of the final pharmacokinetic model. Open circles indicate observations; solid black lines are the lines of identity; red lines are the line of locally weighted scatterplot smoothing.

**Figure 2:**
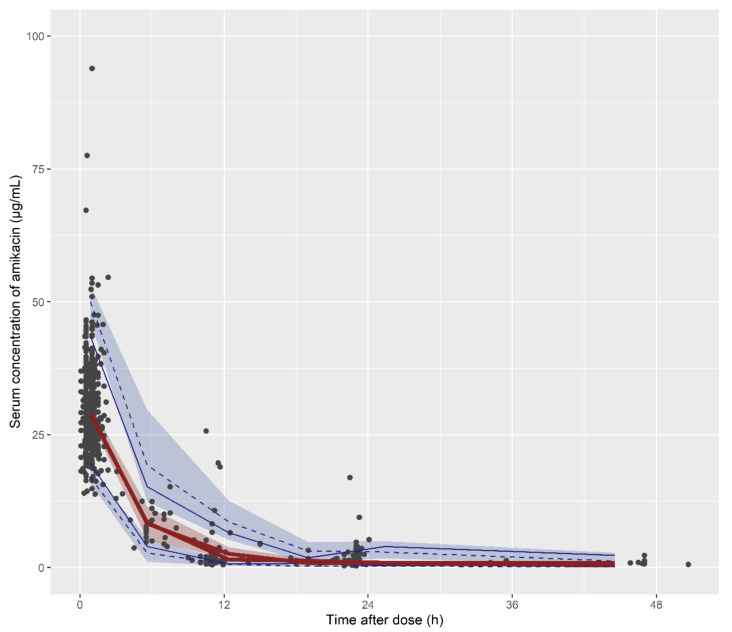
Prediction-corrected visual predictive check for the final population pharmacokinetic model. The solid circles represent the observed amikacin serum concentrations; the solid lines represent the 5th (blue), median (red), and 95th (blue) percentiles of the observed concentration; the dashed lines represent the 5th (blue), median (red), and 95th (blue) percentiles of the simulated concentration; the blue and red areas indicate the 90% confidence interval of the simulated concentrations of each percentile.

A two-compartment model with first-order elimination kinetics provided the best description of amikacin PK. The goodness-of-fit (GOF) plots showed good agreement between the observations and the values, and the conditional weighted residuals (CWRES) were dispersed at approximately zero and did not show any relevant trends, indicating that the amikacin PK characteristics were well described by the final developed model (Figure 1). The prediction-corrected visual predictive check (pc-VPC) plots indicated that the observed data fell well within the 95% prediction intervals of the predicted percentiles and showed good agreement between the observed and the predicted amikacin concentrations (Figure 2). The values estimated through the bootstrap analysis and the final model were similar (Table 2).

In the final model, the inter-individual variabilities (IIVs) for clearance (CL) and central compartment volume (V1) were estimated with omega block to assess the covariance between IIVs for CL and V1. The estimated glomerular filtration rate (eGFR) and body weight were identified as significant covariates for CL and V1, respectively (see Table S1 in the Supplemental Material). The estimated population CL was 3.52 L with 27.9% IIV at the population median eGFR of 91.1 mL/min/1.73 m^2^, and the V1 was 14.4 L with 18% IIV at the population median body weight of 51.1 kg. The eta shrinkages for CL and V1 were estimated as 4% and 21%, respectively. The lowest (29.9 kg) and highest (79.8 kg) body weights obtained from the PK analysis data were used to calculate the V1 of amikacin; the results showed that the V1 of the patient with the highest body weight was 1.99-fold greater than that of the patient with the lowest body weight. Similarly, in terms of the effect of eGFR on the amikacin CL, it was found that patients with the highest eGFR (297.8 mL/min/1.73 m^2^) had a CL that was 1.74-fold greater than that of patients with the lowest eGFR (26.8 mL/min/1.73 m^2^).

### 2.3. Once-Daily Dosing Recommendation

**Figure 3:**
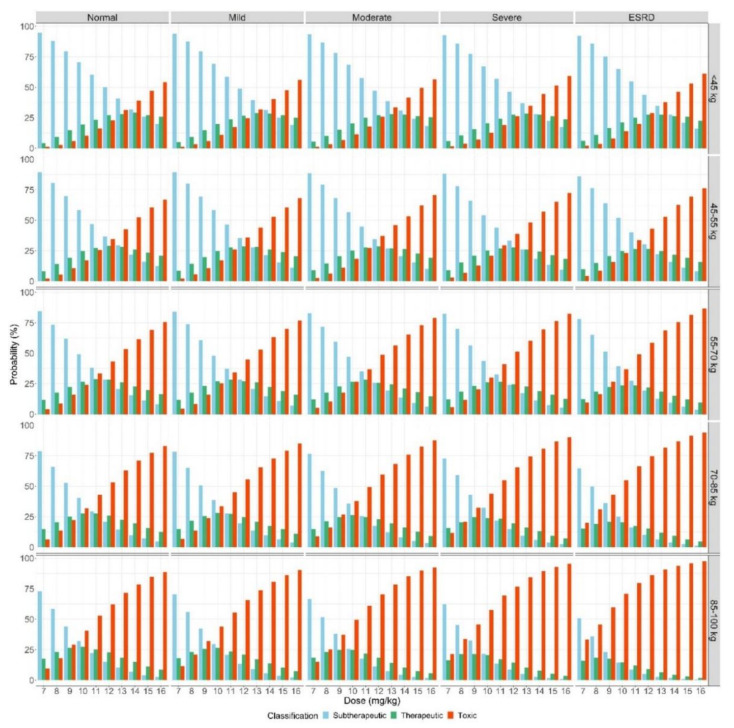
Probability of amikacin therapeutic target attainment from model-based simulations after the following amikacin once-daily dosing regimen on day 5; panel on top of bar plot represents the grade of renal function; Panel on right of bar plot represents the body weight group; Normal, eGFR ≥ 90 mL/min/1.73 m^2^; Mild, 60 ≤ eGFR ≤ 89 mL/min/1.73 m^2^; Moderate, 30 ≤ eGFR ≤ 59 mL/min/1.73 m^2^; Severe, 15 ≤ eGFR ≤ 29 mL/min/1.73 m^2^; ESRD, eGFR < 15 mL/min/1.73 m^2^.

Based on the final model, the concentration-time profiles of amikacin following five intravenous (IV) infusions of various dosages were simulated according to the body weight group and renal function. Then, the optimal dosages of the once-daily dosing regimen were recommended based on the probability of reaching the target therapeutic range at each dose; the dose with the highest target attainment was set as the recommended dose (Figure 3 and Table 3). For patients with a weight less than 70 kg, the optimal once-daily dosage was between 10 and 14 mg/kg, which was within the dosage range recommended by the guidelines. There was no significant difference in once-daily dosage according to renal function. However, patients weighing over 70 kg with kidney disease (e.g., eGFR < 30 mL/min/1.73 m^2^) had a higher probability of reaching target attainment at doses lower than the guideline doses. Therefore, for patients weighing more than 70 kg, renal function should be considered because the eGFR has a greater influence on the amikacin concentration in these patients than in patients with a weight less than 70 kg.

## 3. Discussion

In this study, the optimal pharmacotherapy was explored through analysis of the population PK of amikacin in adult patients diagnosed with NTM-PD. Although amikacin PK have already been studied in populations with various diseases, this is the first study performed in patients with NTM-PD. The treatment of NTM-PD requires the long-term use of antibiotics, and the range of therapeutic concentrations (C_peak_: 35–45 µg/mL, C_trough_: <4 µg/mL) of amikacin is narrower and higher than that necessary for treatment of other indications (C_peak_: <35 µg/mL, C_trough_: <10 µg/mL). As the dose and exposure to amikacin are related to its toxicity, the narrow and high therapeutic concentration range affects the therapeutic effects and causes serious adverse events such as hearing loss and kidney failure [18]. In addition, approximately 20% of patients stop taking amikacin because of side effects [17]. Therefore, it is necessary to identify the pharmacokinetic characteristics of amikacin in patients with NTM-PD and to adjust the dose based on individual characteristics.

In this study, the amikacin concentration-time data were best fit by a two-compartment structure model, while the limited sample collection for determination of the C_peak_ and C_trough_ generally only permits the use of one-compartment models [19,20,21,22,23]. Although our concentration data were also sparsely collected at two sampling points (mainly C_peak_ and C_trough_) for routine therapeutic drug monitoring (TDM), 55 blood samples among the 848 blood samples were collected outside the sampling times corresponding to the C_peak_ and C_trough_. As a result, the two-compartment model showed a significant improvement in GOF over the one-compartment model, which indicated that the two-compartment model is better suited to describe our data.

The population CL and V1 values estimated in this study were similar to those of the previously reported two-compartment model for patients with cystic fibrosis (i.e., 3.06 L/h and 14.4 L for CL and V1, respectively), and were also consistent with known PK properties (i.e., 94 mL/min of CL and 0.21 L/kg of V) [20,21]. Therefore, it is expected that the PK characteristics of amikacin in NTM-PD patients will not be very different from those of patients diagnosed with other diseases. This result is supported by the following pharmacokinetic properties of amikacin: amikacin is hydrophilic; the distribution is mainly restricted to extracellular fluids; amikacin is eliminated through the kidney without metabolism [24]. In addition, since NTM-PD is not a disease that causes weight loss or renal impairment, it is believed that the PK characteristics of amikacin in patients with NTM-PD are not significantly different from those in patients treated for other indications [25].

**Figure 4:**
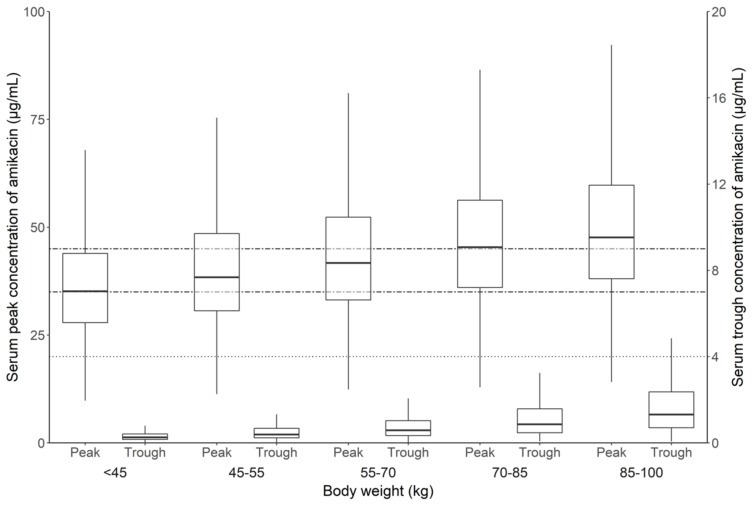
Box plot of predicted amikacin concentration following five times amikacin intravenous administration with 12 mg/kg once daily dosing regimen in various body weight patients with normal renal function. The dotted line represents the therapeutic range of C_trough_ (<4 µg/mL); the dot-dashed lines represent the therapeutic range of C_peak_ (35–45 µg/mL).

In the final model of amikacin PK, body weight was identified as an important covariate that was shown to influence the V1 of amikacin. The association between total body weight and the volume of distribution has already been reported and affects the serum concentration of amikacin [26]. Therefore, dosing according to body weight has been recommended as the conventional dosing method. However, the body weight exponent for V1 in the final model was estimated to be 0.702 in this study, which is less than 1, so V1 is expected to increase less in proportion to the body weight. Accordingly, when the same dosage according to body weight of amikacin is administered to heavier patients, the amikacin concentration is expected to be higher than that in other patients. In our simulation results, the serum concentration of amikacin increased as the body weight increased when 12 mg/kg amikacin was administered once daily to patients with normal renal function (i.e., 90 mL/min/1.73 m^2^) (Figure 4). Body weight-based dosing in obese patients is often a problematic recommendation [27,28]. Therefore, different amikacin dosage recommendations according to the body weight group are required even though the amikacin dosage is determined based on mg/kg.

Not only should body weight be considered in terms of the PK of amikacin, but it is reasonable for eGFR to be a covariate for CL, as amikacin is excreted mainly through the kidneys [29,30]. Moreover, according to the simulation results of this study, the recommended dosage for patients with body weights over 70 kg who have renal impairment should be 8–9 mg/kg, which is less than the dosage of 10–15 mg/kg recommended in the guidelines [4]. Therefore, it is necessary to consider renal function when adjusting the dose, especially for patients with body weights over 70 kg.

In this study, the primary limitation was that the effect of concomitant medications on the PK properties of amikacin were not considered in the study. However, no significant interactions of amikacin with other medications have been reported; therefore, concomitant medications would not be expected to affect the analysis of amikacin PK in the general patient population [8,10]. On the other hand, we only considered the C_peak_/MIC target ratio as an indicator of amikacin efficacy, and explored the optimal dosage in this study. Some papers have suggested that the ratio of area under the concentration–time curve (AUC) to MIC can be used as an indicator of bacterial killing and efficacy of aminoglycosides [31]. However, the AUC/MIC target for amikacin is not clear, and it is more practical and feasible to use C_peak_ and C_trough_ than to calculate AUC in clinical practice. Therefore, we used C_peak_/MIC as a clinically relevant target indicator in this study.

## 4. Materials and Methods

### 4.1. Study Population

Data were collected retrospectively from patients who received IV amikacin and TDM for NTM-PD treatment at the Seoul National University Hospital (SNUH) between 1 December 2009 and 1 December 2019. Patients were included who were administered amikacin for the treatment of NTM-PD, and if both the amikacin dosage and serum concentration–time records were available. Among them, hemodialysis patients were excluded because hemodialysis will affect the PK characteristics of amikacin. This study was reviewed and approved by the Institutional Review Board at SNUH, Seoul, Republic of Korea (IRB No.1912-079-1088).

Amikacin was administered by IV infusion over 30 min or 1 h at intervals of 8, 12, 24, or 48 h. Generally, two blood samples were obtained and used for TDM: one sample was obtained for the C_peak_ measurement, and the other sample was obtained for the C_trough_ measurement. Most of the blood samples for the C_peak_ measurement were obtained within 60 min after the amikacin infusion was completed, and most of the C_trough_ samples were obtained within 30 min prior to the next infusion. Amikacin concentrations below the limit of quantitation were excluded from the analysis. The patient’s age, sex, body weight, height, serum creatinine concentration, eGFR, and serum albumin concentration were also recorded immediately before TDM sampling. The eGFR was computed by using the Modification of Diet in Renal Disease formula [32].

### 4.2. Population Pharmacokinetic Analysis

A population PK analysis was conducted with the logarithmically transformed concentration data using a nonlinear mixed effects method. The first-order conditional estimation method with the interaction option was implemented in NONMEM (version 7.4.3, Icon Development Solutions, Ellicott City, MD, USA). Pirana (version 2.9.6) and RStudio (version 1.2.1335) were used as graphical user interface in the analysis.

One- and two-compartment PK models with linear elimination were tested to determine the model structure. An exponential model was used to estimate the IIV of the PK parameters. An additive model was used to describe the residual error. The selection of the base model was determined by the GOF plots, the precision of the numerical estimates, and the decrease in the objective function value (OFV).

After selection of the base model, the following individual characteristics were evaluated for covariate selection: age, body weight, height, serum creatinine concentration, serum albumin concentration, and eGFR as continuous covariates, and sex as a categorical covariate. For the continuous covariates, both linear (1) and power (2) relationships were tested, whereas categorical covariates were tested using a categorical model (3), as follows:

(1) $$P=\theta_{1}\cdot \left[1+\theta_{2}\cdot \left(\mathrm{COV}-\mathrm{MED}\right)\right]$$

(2) $$P=\theta_{1}\cdot {\left(\frac{\mathrm{COV}}{\mathrm{MED}}\right)}^{\theta_{2}}$$

(3) $$P=\theta_{1}\cdot \left(1+\theta_{2}\cdot \mathrm{IND}\right)$$

where P is the typical individual parameter; θ_1_ is the typical value of P; θ_2_ is the effect of the covariate; COV is the individual covariate value; MED is the median value of the covariate; IND is an indicator variable with a value of either 0 or 1 for categorical covariate values (0: female; 1: male). The significance of the covariates was tested by stepwise forward selection and backward elimination based on the criteria of the OFV with the significance set at *p* < 0.05 and *p* < 0.01, respectively. In addition to the statistical criteria, a decrease in IIV and physiological plausibility were also considered for inclusion of covariates in the model.

### 4.3. Model Evaluation

The developed population PK model of amikacin was evaluated by GOF plots, bootstrap analysis, and a pc-VPC. The GOF plots consisted of the following four plots: observations versus individual predictions; observations versus population predictions; conditional weighted residuals versus population predictions; conditional weighted residuals versus time after dosing. The pc-VPC was used to confirm that the observed data points were overlaid within the median and 90% prediction interval of 1000 simulated datasets from the final model. The bootstrap median values and 95% confidence intervals for each parameter estimate were compared with those estimated from the original dataset.

### 4.4. Optimal Dosing Simulation

Based on the final model, model-based simulations were performed to predict the amikacin concentrations after once daily amikacin dosing for 5 days, which is considered to be a sufficient time to reach the steady state. The simulation was performed for each renal function classification (i.e., eGFR ≥ 90, 60 ≤ eGFR ≤ 89, 30 ≤ eGFR ≤ 59, 15 ≤ eGFR ≤ 29, and eGFR < 15 mL/min/1.73 m^2^) for each body weight group (i.e., weight <45, 45 ≤ weight < 55, 55 ≤ weight < 70, 70 ≤ weight < 85, and weight ≥85 kg) for various once-daily doses (7–16 mg/kg). The optimal therapeutic dose according to the renal function and body weight of the patients was explored based on the probability of reaching the therapeutic range on day 5. The therapeutic range of amikacin was set according to a C_peak_ between 35 and 45 µg/mL and a C_trough_ less than 4 µg/mL, as specified in the amikacin dosing guidelines [10].

## 5. Conclusions

In this study, we analyzed amikacin PK using population PK modeling in Korean patients with NTM-PD. The final population PK model provided a good description of the amikacin PK, and an optimal once-daily dosage of amikacin was suggested to be determined based on the body weight and renal function of the patients. This suggested dosage regimen will provide a rationale to individualize pharmacotherapy and therefore improve the clinical outcomes of amikacin treatment for patients with NTM-PD.

## Figures and Tables

**Table 1:** Demographic characteristics of patients.

Variable	Patients (N = 70)
	Mean ± SD (Min–Max)
Age (years)	66.0 ± 11.5 (25.0–85.0)
BMI (kg/m^2^)	20.2 ± 3.7 (12.8–33.3)
Height (cm)	160.5 ± 8.2 (146.1–177.0)
Body weight (kg)	51.9 ± 11.2 (29.9–79.8)
Serum creatinine (mg/dL)	0.7 ± 0.2 (0.3–1.9)
eGFR (mL/min/1.73 m^2^)	95.7 ± 36.4 (26.8–297.8)
Albumin (g/dL)	3.6 ± 0.5 (1.6–4.8)

SD, standard deviation; BMI, body mass index; eGFR, estimated glomerular filtration rate calculated using Modification of Diet in Renal Disease formula (i.e., eGFR = 175 × Serum creatinine^−1.154^ × Age^−0.203^ × (0.742 if female)).

**Table 2:** Pharmacokinetic and covariate parameters in the base and final population models.

Parameter	Based Model (OFV = −899.01)		Final Model (OFV = −943.678)		Bootstrap (n = 1000)
	Estimate (% RSE)	IIV (% RSE)	Estimate (% RSE)	IIV (% RSE)	Median (95% CI)
CL (L/h)	3.5 (5)	30.4% (10)	3.52 (5)	27.9% (10)	3.527 (3.219–3.789)
~eGFR effect on CL	-	-	0.229 (32)	-	0.228 (0.051–0.331)
V1 (L)	14.5 (4)	21.9% (10)	14.4 (4)	18% (12)	14.297 (13.462–15.250)
~WT effect on V1	-	-	0.702 (17)	-	0.681 (0.472–0.897)
V2 (L)	16.4 (35)	-	14.2 (34)	-	14.864 (8.946–41.629)
Q (L/h)	0.443 (24)	-	0.464 (25)	-	0.477 (0.318–0.685)
Covariance between etas of CL and V1	0.00623	-	−0.0135	-	−0.012 (−0.289–0.006)
Additive residual error	0.303 (8)	-	0.299 (8)	-	0.294 (0.254–0.331)

CL = 3.52 × (eGFR/91.1)^0.229^ × exp (η); V1 = 14.4 × (WT/51.1)^0.702^ × exp (η); V2 = 14.2; Q = 0.464. OFV, objective function value; IIV, inter-individual variability; RSE, relative standard error; CI, confidence interval; CL, clearance; eGFR, estimated glomerular filtration rate; V1, central compartment volume; WT, body weight; V2, peripheral compartment volume; Q, inter-compartmental clearance.

**Table 3:** Once-daily dosage recommendations based on body weight and renal function.

Body Weight (kg)	Normal (≥90 mL/min/1.73 m^2^)	Mild (60–89 mL/min/1.73 m^2^)	Moderate (30–59 mL/min/1.73 m^2^)	Severe (15–29 mL/min/1.73 m^2^)	ESRD (<15 mL/min/1.73 m^2^)
<45	14	13	13	13	13
45 ≤ WT < 55	12	12	12	12	12
55 ≤ WT <70	11	11	11	11	10
70 ≤ WT <85	11	10	10	9	9
WT ≥ 85	10	10	10	9	8

WT: body weight; ESRD: end-stage renal disease. The unit of dose is mg/kg.

## Supplementary Materials

The following are available online at https://www.mdpi.com/2079-6382/9/11/784/s1, Table S1: Sequential covariate model development.
