# Supplementary material for: Population Pharmacokinetic Analysis of Amikacin for Optimal Pharmacotherapy in Korean Patients with Nontuberculous Mycobacterial Pulmonary Disease
Source: Antibiotics (Basel). 2020 Nov 6;9(11):784. doi: 10.3390/antibiotics9110784 (PMC7694782; doi:10.3390/antibiotics9110784)
Supplement: Supplementary file 1 [file antibiotics-09-00784-s001.pdf]

Supplementary Matrerial

Table S1. Sequential covariate model development

Model	Hypothesis	OFV	ΔOFV	Basis of model	% IIV for CL (% RSE)	% IIV for V1 (% RSE)
0	Base model	-899.01	-	-	30.4 (10)	21.9 (11)
1	$TVCL=\theta_1*(eGFR/91.1)^{\theta_2}$	-926.358	-27.348	0	28.3 (10)	22.4 (10)
2	$TVCL=\theta_1*(1+(eGFR/91.1)*\theta_2)$	-918.41	-19.400	0	28.8 (10)	22.3 (10)
3	$TVCL=\theta_1*(WT/51.1)^{\theta_2}$	-905.979	-6.969	0	28.9 (11)	22.0(10)
4	$TVCL=\theta_1*(1+(WT/51.1)*\theta_2)$	-905.426	-6.416	0	29.0 (11)	22.0 (10)
5	$TVCL=\theta_1*(ALB/3)^{\theta_2}$	-924.932	-25.922	0	29.1 (12)	22.2 (10)
6	$TVCL=\theta_1*(1+(ALB/3)*\theta_2)$	-927.422	-28.412	0	29.2 (12)	22.2 (10)
7	$TVCL=\theta_1*(1+(SEX)*\theta_2)$	-902.673	-3.663	0	30.2 (11)	22.0 (10)
8	$TVV1=\theta_1*(eGFR/91.1)^{\theta_2}$	-903.822	-4.812	0	30.4 (10)	22.5 (11)
9	$TVV1=\theta_1*(1+(eGFR/91.1)*\theta_2)$	-902.54	-3.530	0	30.4 (10)	22.1 (10)
10	$TVV1=\theta_1*(WT/51.1)^{\theta_2}$	-921.816	-22.806	0	30.2 (10)	17.5 (11)
11	$TVV1=\theta_1*(1+(WT/51.1)*\theta_2)$	-922.518	-23.508	0	30.4 (10)	22.1 (10)
12	$TVV1=\theta_1*(ALB/3)^{\theta_2}$	-923.684	-24.674	0	30.5 (10)	19.6 (9)
13	$TVV1=\theta_1*(1+(ALB/3)*\theta_2)$	-928.722	-29.712	0	30.5 (10)	19.5 (10)
14	$TVV1=\theta_1*(1+(SEX)*\theta_2)$	-921.869	-22.859	0	30.5 (10)	17.5 (14)
15	$TVCL=\theta_1*(eGFR/91.1)^{\theta_2}$, $TVV1=\theta_3*(WT/51.1)^{\theta_4}$	-946.726	-20.368	1	27.9 (10)	18.0 (12)

ΔOFV: change in objective function value compared to the basis of model; IIV: inter-individual variability; RSE: relative standard error; TV: typical value; CL: clearance; V1: central compartment volume; eGFR: estimated glomerular filtration rate; WT: body weight; ALB: serum albumin; θ_1: typical value of the PK parameter.
